# Development of fluorescence/MR dual-modal manganese-nitrogen-doped carbon nanosheets as an efficient contrast agent for targeted ovarian carcinoma imaging

**DOI:** 10.1186/s12951-020-00736-w

**Published:** 2020-11-30

**Authors:** Cuiping Han, Ting Xie, Keying Wang, Shang Jin, Ke Li, Peipei Dou, Nana Yu, Kai Xu

**Affiliations:** 1 School of Medical Imaging, Xuzhou Medical University, Xuzhou, 221006 China; 2 Department of Radiology, Affiliated Hospital of Xuzhou Medical University, Xuzhou, 221004 China; 3 Department of Medical Imaging, Jinshan Hospital Affiliated To Fudan University, Shnghai, 200540 China

**Keywords:** Manganese, Carbon nanosheets, Tumor targeting, Fluorescence/mr dual-modal nanoprobe

## Abstract

**Background:**

Development of sensitive and specific imaging approaches for the detection of ovarian cancer holds great promise for improving the therapeutic efficacy and the lifespan of the patients.

**Results:**

In this study, manganese-nitrogen doped carbon nanosheets (Mn-N-CNSs) coupled with Anti-HE4 monoclonal antibody (Mn-N-CNSs@Anti-HE4) were synthesized for the specific and targeted fluorescence/MR dual-modal imaging of ovarian carcinoma. The prepared Mn-N-CNSs revealed excellent aqueous dispersity, good colloidal stability, great optical properties and high longtudinal relaxivity rate (r_1_ = 10.30 mM^−1^ s^−1^). Encouraged by the tunable photoluminiscence of the nanoprobe and Anti-HE4 targeting ligand, the ovarian carcinoma cells were specifically labeled by the Mn-N-CNSs@Anti-HE4 nanoprobe with multi-color fluorescences. Benefiting from the high r_1_ relaxivity, the nanoprobe exhibited targeted and enhanced MR contrast effect in the ovarian carcinoma cells and tumor bearing mice model. Besides, the high biocompatibility and easy excretion from the body of the nanoprobe were further confirmed in vivo.

**Conclusion:**

The prepared Mn-N-CNSs@Anti-HE4 with excellent biocompatibility, high-performance and superior tumor-targeting ability provides a novel fluorescence/MR dual-modal nanoprobe for specific labeling and detection of ovarian carcinoma cells in vitro and in vivo.

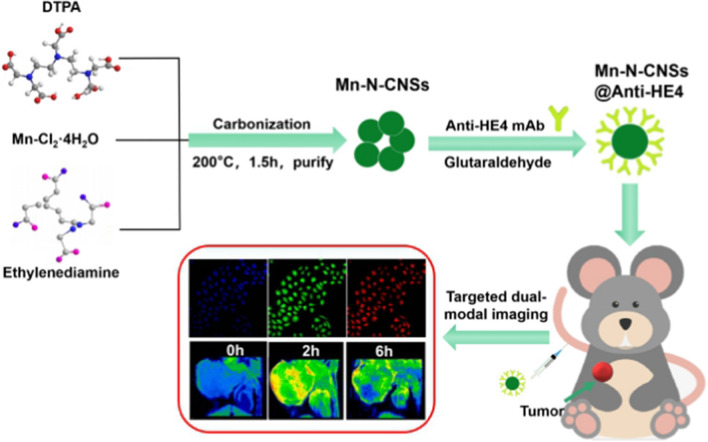

## Background

As reported, ovarian carcinoma is a common and deadly gynecologic cancer with an incidence of approximately 25 ~ 30% in the female genital cancers [1]. The 5-year survival rate was reported to be 92% for the early ovarian carcinoma patients with effective treatments [2]. However, most of ovarian carcinomas have progressed to the advanced stages (FIGO III ~ IV) even with a distant metastasis upon their diagnosis [3, 4]. Therefore, developing a imaging probe to realize early and accurate diagnosis of the ovarian carcinoma is urgently needed for improving the therapeutic efficacy and the lifespan of the patients [5].

Recently, multimodal imaging based on molecular nanoprobe has gained increasing interests for the specific and accurate diagnosis of the tumor [6–9]. It could integrate the merits of different imaging techniques to improve the effectiveness and sensitivity of the diagnosis. Till date, various imaging techniques have been proposed, including magnetic resonance imaging (MRI), fluorescence imaging (FL), computed tomography imaging (CT), position emission tomography (PET) and photoacoustic imaging (PA), etc. [6, 7]. Among these imaging methods, MR imaging is a powerful technique for high spatially resolved visualization of the anatomic structure and function of soft tissues with a deep penetration, but it has the disadvantage of low sensitivity [8–10]. Fluorescence imaging, as a functional imaging method, could compensate the limitation of MR imaging for its high sensitivity in cellular and subcellular levels [10–12]. Therefore, the combination of MRI and FL imaging renders a promising dual-modal imaging technique with complementary characteristics to improve the accuracy of tumor diagnosis [11–13].

In recent years, manganese (Mn) offers great advantages as a T_1_-weighted MR contrast agent attributed to the presence of five unpaired electron in the bivalent state [14, 15]. Unlike the lanthanides including Gd, the non-lanthanide metal Mn is a natural cellular consistent, and usually acts as a cofactor for various enzymes and receptors [15]. However, it has been reported that the free ions of Mn could induce obvious toxicity to the neuron system of mice and rats after intravenous and intraperitoneal administration [15, 16]. Therefore, the formulation of Mn is needed to prohibit the leakage and exposure of Mn ions to the biological conditions. Various nanomaterials were used to address this problem, including micelles, chelated Mn, liposomes and carbon nanomaterials, etc. [17–20].

Over the past decades, carbon nanomaterials have been widely researched in biomedical applications attributed to their remarkable optical property, good aqueous dispersity, low toxicity and excellent biocompatibility, including carbon nanotubes, nanodiamond, fullerene C_60_, graphene oxide, carbon nanodots, carbon nanosheets [21]. Encouraged by these properties, carbon nanomaterials have been applied in various biomedical fields, including bioimaging, biosensing, cell labeling, etc. [22, 23]. In particular, heteroatom-doped carbon nanomaterials draw great attention among researchers since heteroatoms doping can effectively modulate the surface defects and local chemical features for special bio-applications [24]. The N atom, having a comparable atomic size and five valence electrons for bonding with carbon atoms, has been widely used for chemical doping of carbon nanomaterials [25, 26]. Besides the excellent optical property, carbon nanosheets also possess the wonderful ability and large surface to embed metallic atoms and chemotherapeutic drugs for MR imaging and drug delivery, respectively [27–29]. For example, Chen et al. synthesized Gd-encapsulated carbonaceous nanosheets with for magnetic resonance imaging with efficient renal clearance [27]. Du et al. prepared engineered Gd-doped carbon nanosheets with a high relaxivity for magnetic resonance imaging-guided radiotherapy for tumors [29]. The 2D nanosheets structure could provide more surface to load the Gd or Mn ions, thus enhancing their MR contrast ability.

Although the multi-modal molecular nanoprobe holds many advantages in tumor imaging, it may fail to accurately diagnose the tumor tissues owing to the low specificity of the nanoprobe to different tumors [30–33]. Therefore, various tumor targeting ligands have been used to modified the molecular nanoprobes to improve their target ability to tumors, including antibodies, proteins folate acid and RGD, etc. [30–34]. Recently, human epididymis protein 4 (HE4) has been detected in the epithelium of the distal epididymis, which is also found commonly over-expressed in ovarian cancer [35]. It is reported that the HE4 exhibited higher sensitivity to the ovarian cancer compared to the conventional CA125 biomarker especially in the early stages. Therefore, the HE4 has been identified as a new biomarker for the early diagnosis of ovarian carcinoma [36].

**Scheme. 1:**
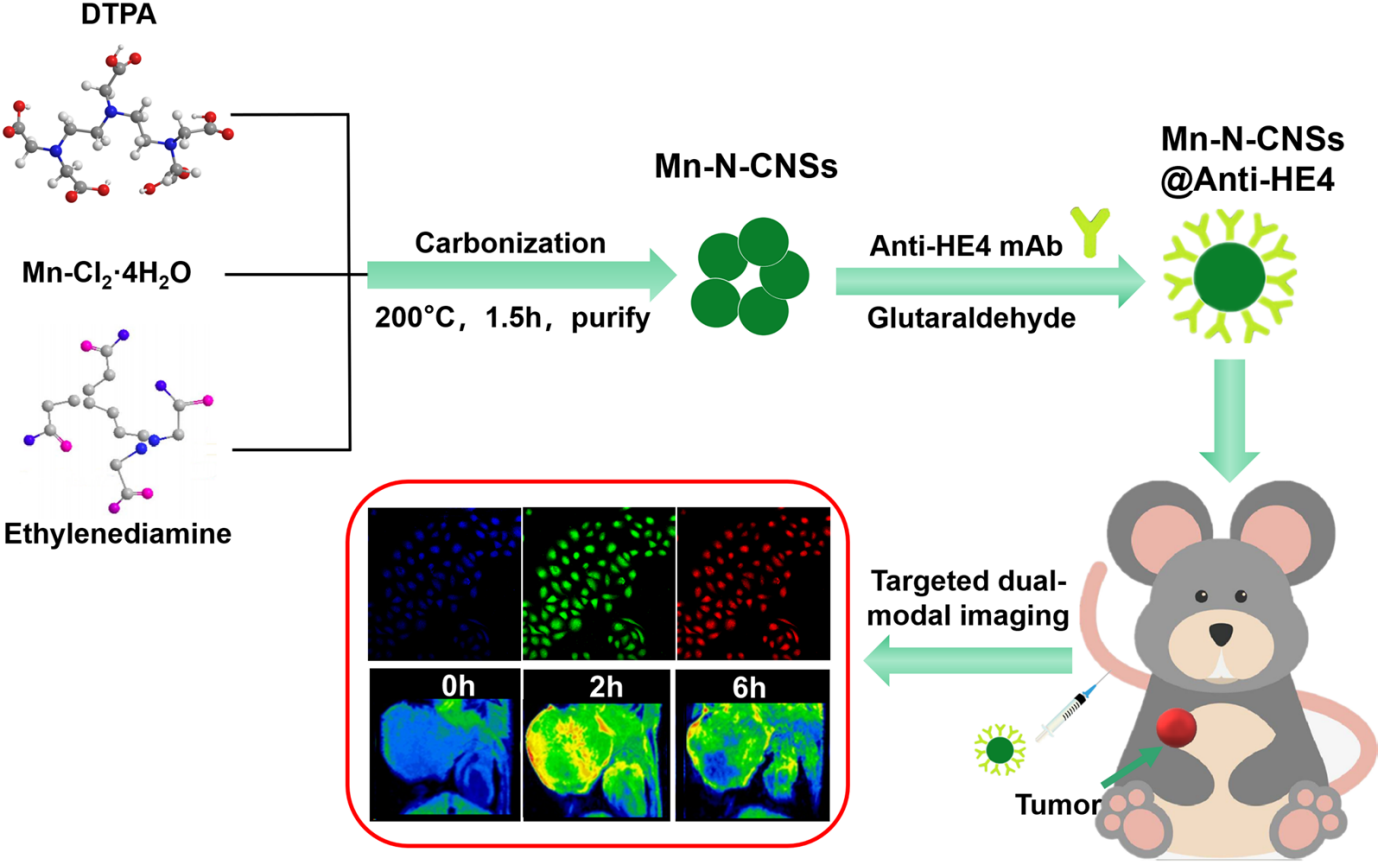
Schematic illustration of Mn-N-CNSs and Mn-N-CNSs@Anti-HE4 formation process and tumor targeted FL/MR dual-modal imaging

Herein, novel manganese and nitrogen atoms co-doped carbon nanosheets (Mn-N-CNSs) were developed for the FL/MR dual-modal imaging of ovarian carcinoma, as illustrated in Scheme 1. Anti-HE4 mAb conjugation was applied to improve the affinity of the nanoprobe to the ovarian carcinoma cells, thus, enhancing its target ability in vitro and in vivo. Taking advantage of the excellent inherent fluorescence property, multi-color specific fluorescence imaging potential of Mn-N-CNSs@Anti-HE4 nanoprobe on HO-8910 ovarian carcinoma cells was achieved. More importantly, the nanoprobe showed high longtudinal relaxivity and further deployed for the in vitro MR imaging for HO-8910 ovarian carcinoma cells and in vivo MR imaging on the tumor bearing mice. In addition, in vitro cell viability and in vivo serum biochemistry and histological analysis were also performed to evaluate the biocompatibility of the nanoprobe.

## Materials and methods

### Materials

Diethylenetriaminepentaacetic acid (DTPA), manganese chloride tetrahydrate (MnCl_2_•4H_2_O), ethylenediamine and acetone were purchased from Sinopharm Chemical Reagents Co. Ltd. (Shanghai, China). Magnevist (Gd-DTPA) was obtained from Bayer Schering Pharma AG (Berlin, Germany). Concentrated nitric acid was received from Zhongtai Chemical Reagents Co. Ltd. (Shanghai, China). Dimethyl sulfoxide (DMSO), thiazolyl blue tetrazolium bromide (MTT) was received from Sigma-Aldrich Co. (St. Louis, MO, USA). Anti-HE4 monoclonal antibody was supplied by the Abcam (Shanghai) Trading Ltd. Roswell Park Memorial Institute 1640 Medium (RPMI-1640), fetal bovine serum (FBS), PBS buffer, EDTA-trypsin, and penicillin/streptomycin were purchased from Gibco BRL (MD, USA). All other chemicals were used in this study were analytical grade.

### Synthesis of manganese-nitrogen doped carbon nanosheets (Mn-N-CNSs)

The Mn-N-CNSs were synthesized via the high-temperature pyrolysis method using DTPA, MnCl_2_•4H_2_O and ethylenediamine as the carbon, manganese and nitrogen sources, respectively. Briefly, DTPA (0.5 mmol) and MnCl_2_•4H_2_O (0.5 mmol) was dissolved in 40 mL deionized water and stirred for 1 h under 500 rpm at 55 °C to form transparent homogeneous solution. Then, the mixing solutions (1 mL) were added with ethylenediamine (0.01 mmol) and were heated 200 °C for 1.5 h. Subsequently, the resultant solutions were re-dispersed in deionized water and washed thrice with acetone by centrifuging at 10,000 rpm for 5 min to obtain Mn-N-CNSs.

The Anti-HE4 mAb was conjugated on the Mn-N-CNSs through a glutaraldehyde cross-linking procedure. Briefly, glutaraldehyde (100 μL, 5%, w/v) were added to the Mn-N-CNSs sodium borate buffer solution (500 μL, 8 mg/mL) and under a stirring condition at room temperature for 1 h. Then, the Anti-HE4 mAb (100 µL, 1 mg/mL) was added in the above solution and stirred for another 1 h. Thereafter, sodium borohydride was added in an ice bath for 1 h, and maintained at 4 °C overnight. The product was centrifuged to remove the excess antibodies and dispersed in PBS for further use.

### Characterization of Mn-N-CNSs

The quantum yield (QY) of the Mn-N-CNSs was evaluated by comparing with the quinine sulfate in H_2_SO_4_ as reference standard. The size and morphology of as-prepared Mn-N-CNSs were recorded by a high resolution-transmission electron microscopy (HR-TEM, FEI Tecnai G2 Spirit). The TEM samples were prepared by dropping the Mn-N-CNSs aqueous suspension on the carbon-coated copper grids. The hydrodynamic diameter and zeta potential were analyzed by a dynamic light scattering analyzer (Zetasizer Nano ZS90, England). UV–vis absorption spectra were obtained using an UV–Vis-NIR spectrometer (UH4150 HITACH). The fluorescence measurements were carried out using a LS-45/55 Fluorescence/Phosphorescence Spectrometer (PerkinElmer, America). The crystal structure of Mn-N-CNSs was investigated by X-ray diffraction (XRD) patterns with a Rigaku diffractometer. The chemical structure of Mn-N-CNSs was recorded with a Fourier Transform Infrared spectrometer (FTIR, Bruker Tensor27) and X-ray photoelectron spectroscopy (XPS, Thermo escalab250Xi, USA). The content of Mn in Mn-N-CNSs was obtained using an Inductively coupled plasma-Mass Spectrometry (ICP-MS, Agilent ICPMS7800).

### Cell culture

HO-8910 human ovarian carcinoma cells and NIH3T3 fibroblast cells were purchased from Cell Bank of the Chinese Academy of Science (Shanghai, China). The above-mentioned cell lines were cultured in RPMI-1640 medium supplemented with 10% (v/v) FBS and 1% penicillin–streptomycin, and maintained in 37 °C in a humidified 5% CO_2_ atmosphere.

### Cell cytotoxicity assessment

The cell cytotoxicity of the Mn-N-CNSs was evaluated by MTT assay [37]. The HO-8910 and NIH3T3 cells were cultured in RPMI-1640 medium for 24 h for the cell attachment. Subsequently, the culture medium was replaced with 100 µL of fresh RPMI-1640 medium containing different concentrations of Mn-N-CNSs (0, 0.02, 0.05, 0.1, 0.2, 0.3, 0.5, 0.75, 1.0 mg/mL), and the cells were incubated for another 24 h. Then, after the medium was replaced with fresh RPMI-1640, MTT solution (10 µL, 5 mg/mL in PBS) was added to each well. After 4 h incubation, the medium was removed, following with the addition of DMSO (100 µL). The absorbance at 570 nm was evaluated using a microplate reader (BioTek Epoch, Service Card). The relative cell viability compared control groups was calculated as a percentage of surviving cells as the mean values of triplicate measurements.

### Cellular uptake experiments

The cells were cultured in RPMI-1640 medium for 24 h. Then, Mn-N-CNSs (2 mM) and Mn-N-CNSs@Anti-HE4 (2 mM) were added to the cells and incubated for another 2 h at 37 °C. After being washed three times with PBS, the cells were detached by incubation with 0.25% trypsin, and collected by centrifugation at 5000 r min^−1^ for 2 min. Afterwards, the cells were fixed by 2.5% glutaraldehyde solution, washed with PBS, dehydrate with graded ethanol and propylene oxide, embedded in Epon, and then dried in an oven at 60 °C for 48 h. Ultrathin slices of approximately 50 nm thickness were obtained using a Leica ultramicrotome (Leica, EM UC7, Germany) with a diamond knife, and the images of the slices were viewed on a Tecnai G2 Spirit BioTWIN transmission electron microscope.

### Cell labeling and fluorescence imaging

The HO-8910 and NIH3T3 cells were deployed to investigate the ability of Mn-N-CNSs and Mn-N-CNSs@Anti-HE4 as targeted fluorescence probe for cancer cell labeling. Cells were seeded in a flat-bottom 12-well plate with glass coverslips in 1 mL culture medium. After overnight incubation, the cells were treated with Mn-N-CNSs (2 mM) and Mn-N-CNSs@Anti-HE4 (2 mM) for cellular uptake. Then, the cells were washed thrice with PBS buffer to remove the excess nanoparticles. Finally, fluorescence imaging was performed using a fluorescence microscopy.

### T_1_-weighted relaxivity measurement

The *T*_1_-weighted relaxivity measurements of Mn-N-CNSs@Anti-HE4 were carried out on a 3.0 T GE Discovery 750 W MR system. The Mn-N-CNSs@Anti-HE4 aqueous solution with various Mn concentration (0.12, 0.36, 0.48, 0.60, 0.84, 0.96 and 1.20 mM, measured by ICP-AES) were prepared. The *T*_1_-weighted relaxivity value of Mn-N-CNSs@Anti-HE4 was obtained by measuring *T*_*1*_ relaxation time as a function of Mn concentration. The parameters of as follows: (i) T1-weighted spin-echo sequence: TR = 425 ms, TE = Min Full, Matrix size = 384 × 224, Field of View = 18 cm × 18 cm, Slice thickness = 3.0 mm, spacing = 1.5 mm; (ii) T1-map images: TE = 7.4 ms, TR = 200–800 ms, FOV = 14 cm × 14 cm, matrix = 384 × 256, slice thickness = 2.0 mm, spacing = 1.5 mm. The T1-map images were generated by function software at a workstation (GE AW 4.6). The mean T1 value of each region of interest (ROI) was calculated.

For comparison, the *T*_1_-weighted relaxivity value of a commercial contrast agent Magnevist (Gd-DTPA) was detected using the same procedures stated above.

### Targeted MR cell imaging

HO-8910 and NIH3T3 cells were cultured on 6-well plate overnight to investigate the MR cell imaging of Mn-N-CNSs. Mn-N-CNSs (2 mM) and Mn-N-CNSs@Anti-HE4 (2 mM) were then co-incubated with the above cells for 2 h. After washing with PBS for three times and trypsinized with EDTA-trypsin, the cells were re-dispersed in 200µL 1% agarose gel. Finally, the MR cell imaging was performed with on a 3.0 T GE Discovery 750 W MR system. The T1-weighted pseudo-color images were generated by function software at a workstation (GE AW 4.6). The ROIs overlying cell pellets were selected in the subsequent data analysis. Signal intensity were derived respectively from T1-weighted pseudo-color images by means of ROI measurements of cell pellets (55 pixels).

### In vivo targeted MR imaging

In vivo MR tumor imaging was performed on the 7-week old female Kunming mice. The xenograft tumor model was established by subcutaneous injection of HO-8910 cells (2 × 10^6^ in 100 µL) on the back. Subsequently, the tumor models were injected via tail vein with Mn-N-CNSs and Mn-N-CNSs@Anti-HE4 (Mn concentration = 4 mg/kg) when the tumor reached 1 cm^3^. MR imaging was conducted pre- and post-injection (0, 15 min, 30 min, 45 min, 1 h, 2 h, 4 h, 6 h, 12 h and 24 h).

### In vivo long-term toxicity study

Kunming mice (female, 7 weeks) were randomly divided into 2 groups (n = 15). The mice were injected via tail vein with PBS and Mn-N-CNSs@Anti-HE4 (Mn concentration = 2, 4 mg/kg). The body weight changes of the injected mice were monitored at 0, 1, 2, 3, 4, 5, 7, 11, 14, and 21d. In addition, the mice were dissected to obtain the major organs (heart, liver, spleen, kidney and lung) at 1, 8 and 22 d post injection (n = 3). Thereafter, the collected organs were fixed with 10% formalin, sectioned and stained with H&E staining. Finally, the histological samples were observed using an optical microscope.

### Statistics analysis

SPSS16.0 software was employed for statistical analysis. Student’s *t*-tests were used to compare the statistical significance between different groups. The data were represented as mean with SD, and p < 0.05 was considered with statistical significant.

## Results and discussions

### Characterization of Mn-N-CNSs

**Fig. 1:**
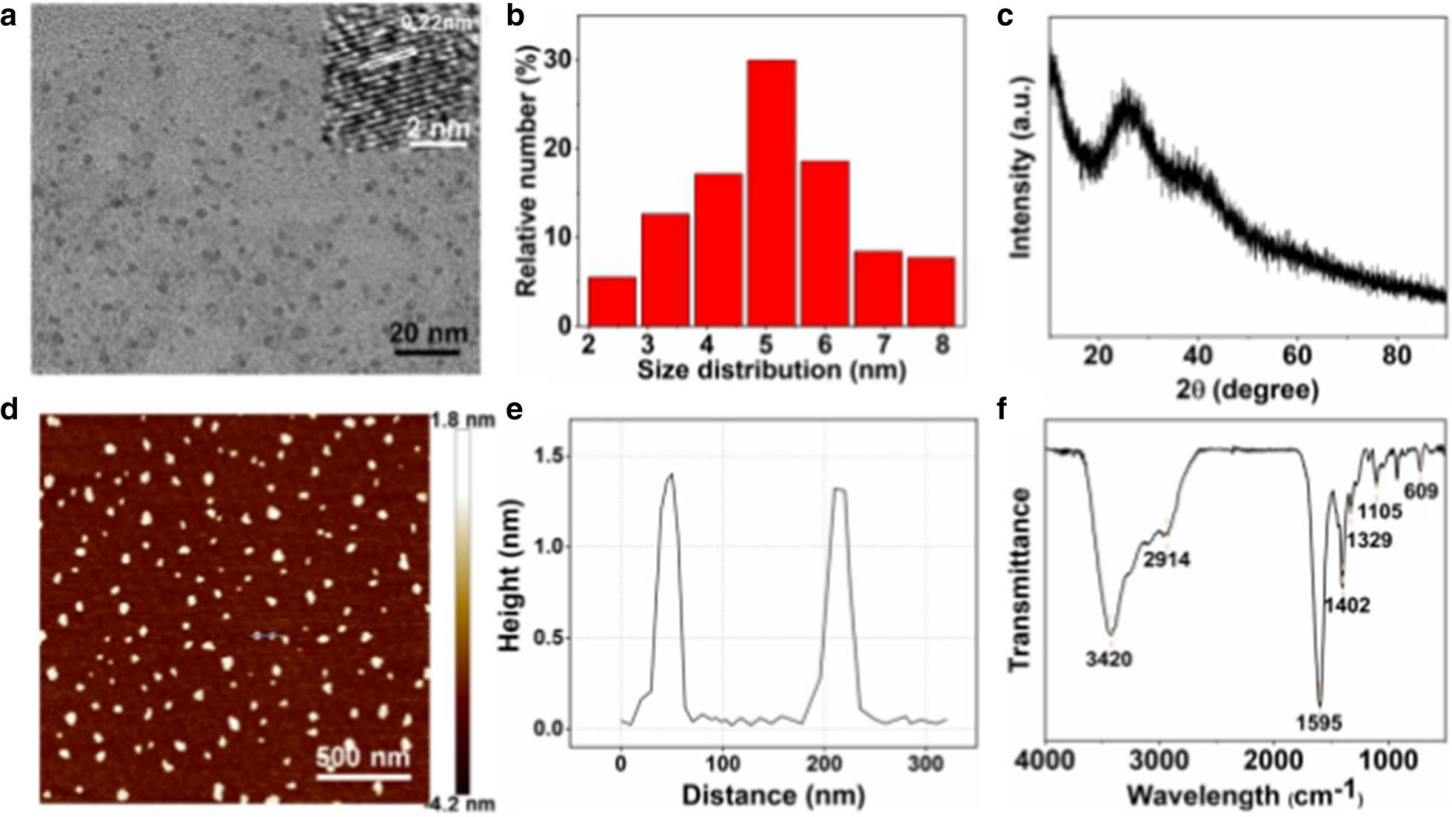
Characterizations of Mn-N-CNSs: **a** TEM images of Mn-N-CNSs, (inset, high resolution TEM images), **b** Size distributions of Mn-N-CNSs, **c** XRD patterns of the Mn-N-CNSs, **d** AFM image of Mn-N-CNSs, **e** Size and height distribution of Mn-N-CNSs, **f** FTIR spectra of the Mn-N-CNSs

The Mn-N-CNSs was prepared via the high-temperature pyrolysis method using DTPA, MnCl_2_•4H_2_O and ethylenediamine as the C, Mn and N sources, respectively. Control experiments showed that the reaction time, temperature, and Mn^2+^ dosage have a great influence on the fluorescence and MRI properties of Mn-N-CNSs ( Additional file 1: Fig. S1). A optimal reaction condition via the carbonization DTPA and MnCl_2_•4H_2_O with the molar ratio of 1:1 in the presence of ethylenediamine reaction at 200 °C for 90 min was established to prepare Mn-N-CNSs with high quality. The morphology of as-synthesized Mn-N-CNSs was imaged by HR-TEM and shown in Fig. 1a. The Mn-N-CNSs revealed a highly uniform and monodispese spherical morphology and an mean diameter in a narrow distribution was measured to be 5.2 nm (Fig. 1b). The high-resolved TEM image exhibited the good crystal structure of Mn-N-CNSs with apparent lattice fringes (Fig. 1a inset), which the lattice fringe of 0.22 nm in the HR-TEM graph was consistent with that of graphene nanosheets [38]. A typical energy dispersive X-ray (EDX) pattern of the nanoparticles revealed that the Mn-N-CNSs consist of C, N, O and Mn elements (Additional file 1: Fig.S2). The content of Mn in Mn-N-CNSs was about 10.2% (wt %), which is higher than that of the previous reported Mn-doped carbon dots [20]. Furthermore, AFM was used to characterize the thickness and morphology of Mn-N-CNSs (Fig. 1d, e). The thickness ranged from 0.5 to 1.4 nm, indicating the monolayer or bilayer structure of Mn-N-CNSs.

The composition and structure of Mn-N-CNSs was also investigated by the XRD, FTIR and XPS analysis. The XRD image shown in Fig. 1c indicated a broad diffraction peak at 28° representing the (002) plane, which was in agreement with that of the two-dimensional carbon nanomaterial, graphene [39]. The FTIR spectra shows the peak at 3420 cm^−1^ for O–H/N–H stretching vibrations, 2914 cm^−1^ and 1105 cm^−1^ for C-H stretching vibrations, 1595 cm^−1^ for C = O stretching vibrations, 1402 cm^−1^ for C = C stretching vibrations, and 1329 cm^−1^ for C-N stretching vibrations (Fig. 1f). Besides, the peak at 609 cm^−1^ corresponding to the Mn–O and Mn-N stretching vibrations represented the coordination absorption peak of Mn^2+^ ions with the carbon nanosheets.

**Fig. 2:**
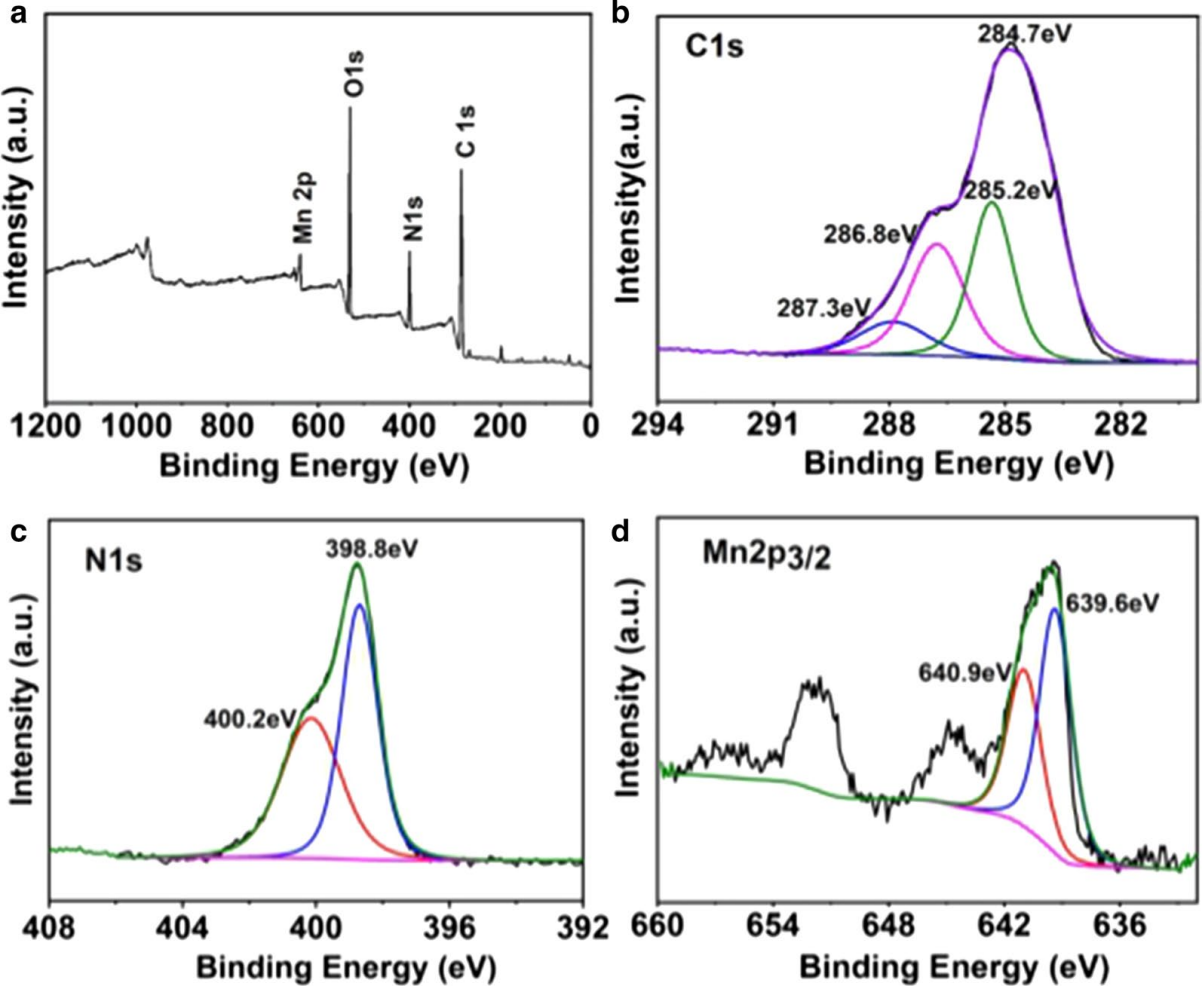
XPS spectra of the Mn-N-CNSs: **a** Full-scan spectrum, **b** C1s expanded spectra, **c** N1s expanded spectra, **d** Mn2p_2/3_ expanded spectra

XPS analysis was performed to analyze the surface elements of Mn-N-CNSs. As exhibited in the XPS spectrum graph (Fig. 2a), the Mn-N-CNSs nanoprobe indicated the presence of C, O, N and Mn on its surface. In the expanded XPS spectra, the C1s peak appeared at 284.7 eV, 285.2 eV and 287.3 eV, corresponded to sp^2^ C in graphene, sp^3^ C in C-O and C-N, and C = O from carbonyls and carboxylates, respectively (Fig. 2b). Two typical binding peaks in N1s spectrum were observed at 400.2 eV and 401.7 eV, indicating the existence of (C)_3_-N and N–H for the nitrogen element, respectively (Fig. 2c). More importantly, two peaks at 640.9 eV and 639.6 eV corresponding to the spin–orbit coupled Mn 2p3/2, suggest that the valence state of Mn in Mn-N-CNSs was Mn^2+^ and Mn^3+^, with the Mn^2+^ in a higher percentage (Fig. 2d). Therefore, these results confirmed the successful synthesis of Mn-N-CNSs from multiple perspectives.

### Optical features of Mn-N-CNSs

**Fig. 3:**
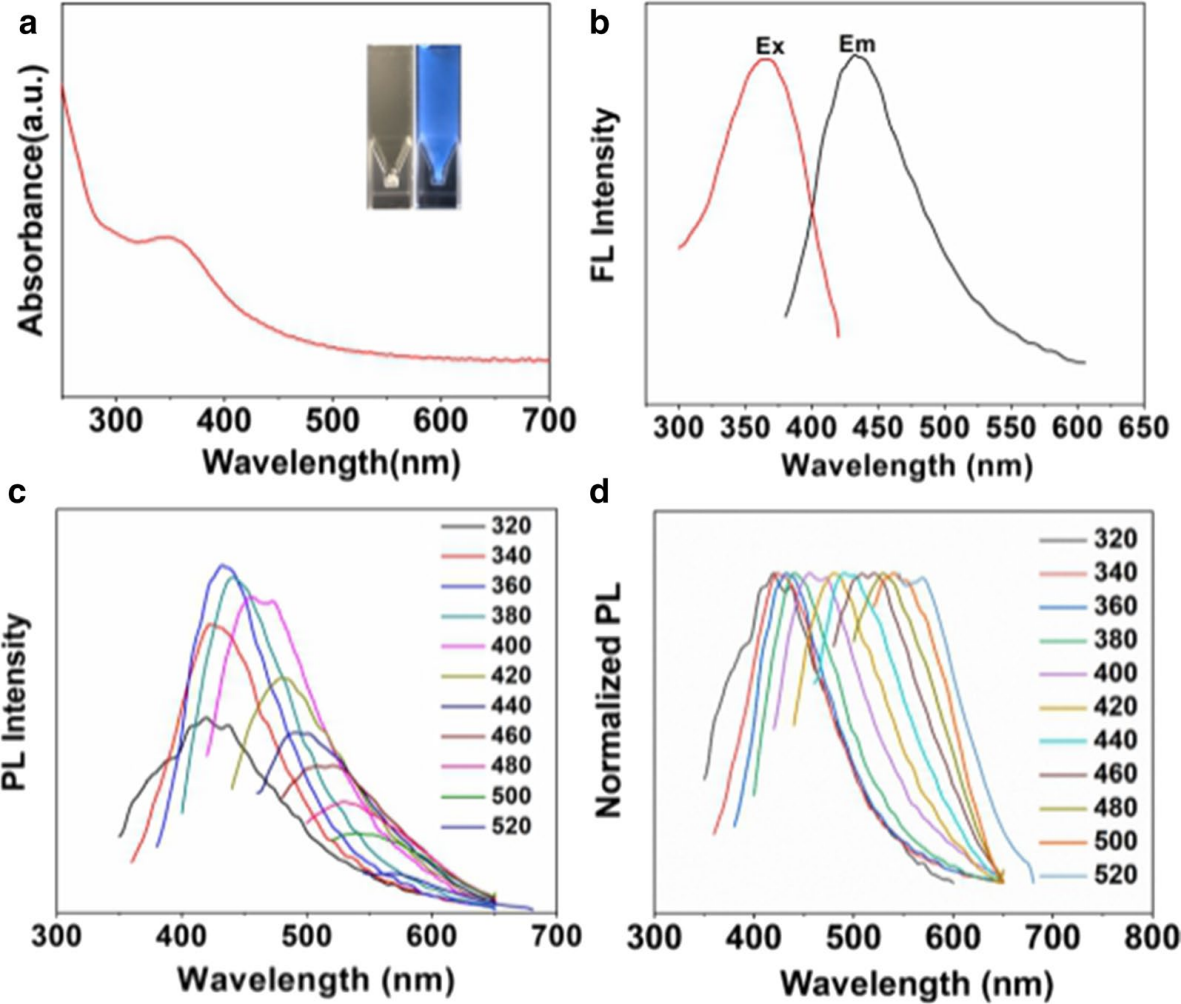
Optical features of Mn-N-CNSs: **a** UV–visible absorption of the Mn-N-CNSs, inset pictures show the Mn-N-CNSs under natural light and UV light, **b** PL emission and excitation of the Mn-N-CNSs, **c** The PL emission of the Mn-N-CNSs with excitation wavelengths from 320 to 520 nm in 20 nm increments, **d** The normalized PL emission of the Mn-N-CNSs with excitation wavelengths from 320 to 520 nm in 20 nm increments

The UV–vis spectrum of Mn-N-CNSs aqueous solution indicated a peak located at 350 nm (Fig. 3a). The Mn-N-CNSs aqueous solution exhibited pale yellow and transparent in daylight, but turned to strong blue color under UV light (365 nm). The fluorescence quantum yield of Mn-N-CNSs solution was investigated and measured to be 52.53% by using quinine sulfate as standard, which is higher than that of Mn-doped carbon quantum dots (13%) [40]. Besides, the Fig. 3 indicated the fluorescence of Mn-N-CNSs at different excitation wavelengths from 320 ~ 520 nm. The PL emission peaks red-shifted from 420 to 580 nm at the excitation wavelength from 320 to 520 nm with the highest fluorescence intensity (435 nm) at the the excitation wavelength of 360 nm, indicating an excitation-dependent emission and good multi-color emission.

### T_1_ longitudinal relaxivity

**Fig. 4:**
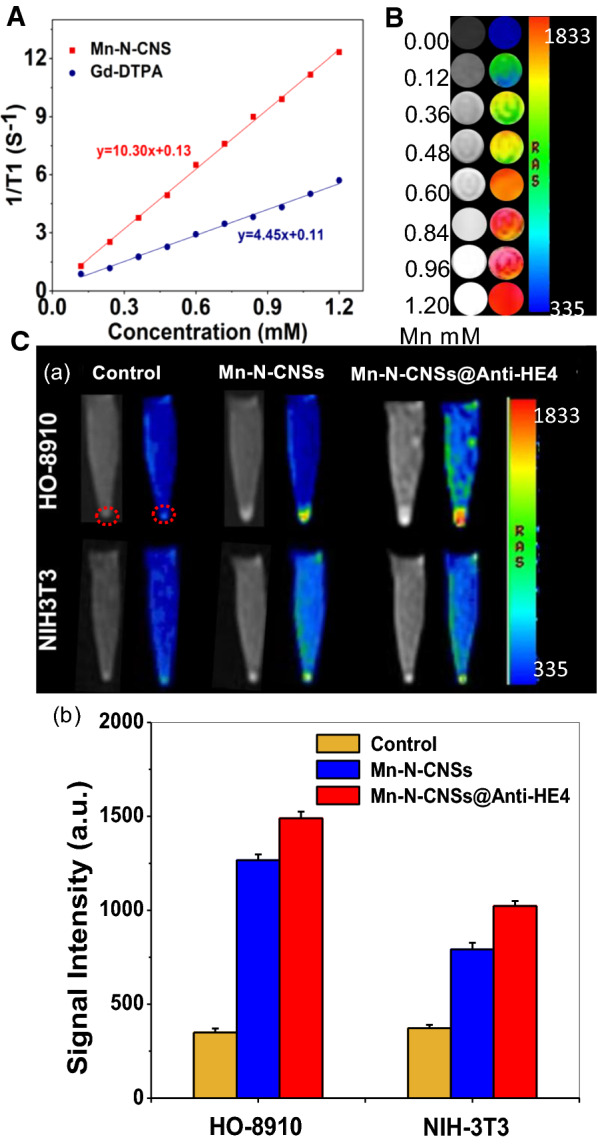
MR contrast ability of samples: **A** Linear relationship between longitudinal relaxation rate of Mn-N-CNSs and Gd-DTPA at different concentrations. **B** T_1_-weighted and corresponding pseudo-color images of Mn-N-CNSs at different Mn concentrations. **C** MR images of HO-8910 and NIH3T3 cells: (**a**) T_1_-weighted and corresponding pseudo-color images, and (**b**) MR signal intensity of cells before and after treated with Mn-N-CNSs@Anti-HE4 (Mn: 2 mM) and Mn-N-CNSs (Mn: 2 mM) for 2 h. The inside of the red circles are cell pellets

Mn-related nanomaterials have emerged as a novel T_1_ weighted contrast agents for MR imaging owing to favorable electronic configuration and enriched biomedical features [41, 42]. As MRI nanoprobe, the MRI behavior of Mn-N-CNSs was investigated compared with the commercial agent Gd-DTPA by the previous 3.0 T MR system (Fig. 4). The relaxivity values of Mn-N-CNSs and Gd-DTPA were detected and compared by measuring the longitudinal relaxation time (T_1_) at various Mn/Gd concentrations. As indicated in Fig. 4B, Mn-N-CNSs clearly induced a concentration dependent brightening effect to the T_1_-weighted MR images, which the bright signal could be enhanced with the increasing concentration of the nanoparticles. The longitudinal relaxation rate (r_1_), obtained by measuring the relaxation time as a function of Mn concentration, was found to be 10.30 mM^−1^ s^−1^, which was 2.3-fold that of the commercial contrast agent Gd-DTPA (4.45 mM^−1^ s^−1^), and much higher than Mn-carbon dots hybrid (3.26 mM^−1^ s^−1^) that we have reported [20]. Such high r_1_ relaxivity value may come from high loading concentration of Mn ions on the surface of carbon nanosheets to shorten the longitunidal relaxation of water protons, thus increasing the signal intensity of T_1_-weighted MR images.

### Stability of Mn-N-CNSs

The stability of Mn-N-CNSs in different solutions including DI water, PBS, FBS and RPMI-1640 medium were investigated. The as-prepared Mn-N-CNSs showed excellent colloidal dispersity in the above mentioned media (Additional file 1: Fig. S3A). The results of dynamic light scattering (DLS) measurement illustrated that the particles had negligible aggregation in the biological fluids (Additional file 1: Fig. S3B). Moreover, the Mn-N-CNSs also displayed stable T1-weighted MR signal in water and different biological fluids, indicating their high stability as MR contrast agent in various media (Additional file 1: Fig. S3C).

The fluorescent stability of Mn-N-CNSs was also investigated under various conditions. The results showed that Mn-N-CNSs maintained high fluorescent stability for a long period (2 month) (Additional file 1: Fig. S4A). The fluorescence anti-photobleaching test also confirmed the excellent fluorescent stability of Mn-N-CNSs under long-time irradiation by UV light (365 nm) (Additional file 1: Fig. S4B). In addtion, the fluorescence intensity of Mn-N-CNSs almost did not change in NaCl solution with high concentration (1 M) (Additional file 1: Fig. S5A) and relatively neutral pH (pH = 5 ~ 9) (Additional file 1: Fig. S5B). All these features make the as-prepared Mn-N-CNSs excellent candidates as a powerful molecular imaging probe for biological application.

### In vitro cytotoxicity and cellular uptake assays

**Fig. 5:**
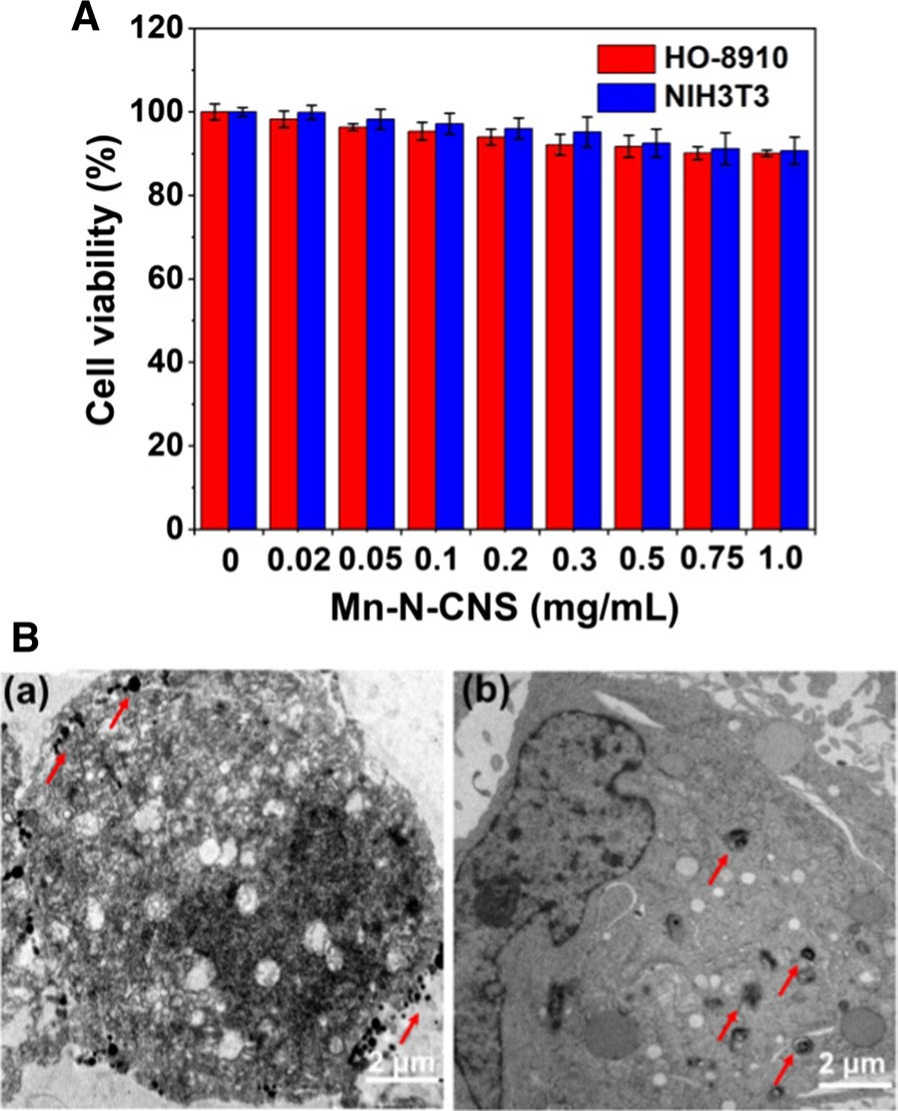
**A** In vitro cytotoxicity tests of Mn-N-CNSs against HO-8910 ovarian carcinoma cell and NIH3T3 cells, **B** Cellular uptake test with Bio-TEM of Mn-N-CNSs@Anti-HE4 by (**a**) NIH3T3 cells and (**b**) HO-8910 cells. The arrows indicate the nanoparticles locations

The cytotoxicity evaluation of the Mn-N-CNSs is critical to ensure the biocompatibility of the nanoprobe to be deployed in the tumor imaging. The in vitro cytotoxicity of Mn-N-CNSs at various concentrations was investigated by performing MTT assays for 24 h against HO-8910 ovarian carcinoma cells and NIH3T3 fibroblast cells (Fig. 5a). The cell viability remained more than 90% at all treated concentrations for two cell lines. Even at the highest concentration of 1 mg/mL (3.2 mM Mn, measured by ICP-MS), 90.1% and 91% of the cells survived after 24 h incubation of the Mn-N-CNSs for the HO-8910 and NIH3T3 cells, respectively, demonstrating the good biocompatibility of Mn-N-CNSs as MRI nanoprobe.

To observe the cellular uptake of Mn-N-CNSs@Anti-HE4 by NIH3T3 and HO-8910 cells, a biological TEM (Bio-TEM) analysis was performed. As Fig. 5b shows, large amounts of Mn-N-CNSs@Anti-HE4 nanoparticles existed outside of the NIH3T3 cell membrane indicating the failed uptake by NIH3T3 cells. However, for the HO-8910 cells, the Mn-N-CNSs@Anti-HE4 nanoprobes were largely phagocytized inside the cells and existed in the intracellular endosomes and the cytoplasm. The result indicated that the Mn-N-CNSs@Anti-HE4 nanoprobes could be selectively taken up and internalized by the HO-8910 ovarian cancer cells.

### In vitro specific cell fluorescence imaging

**Fig. 6:**
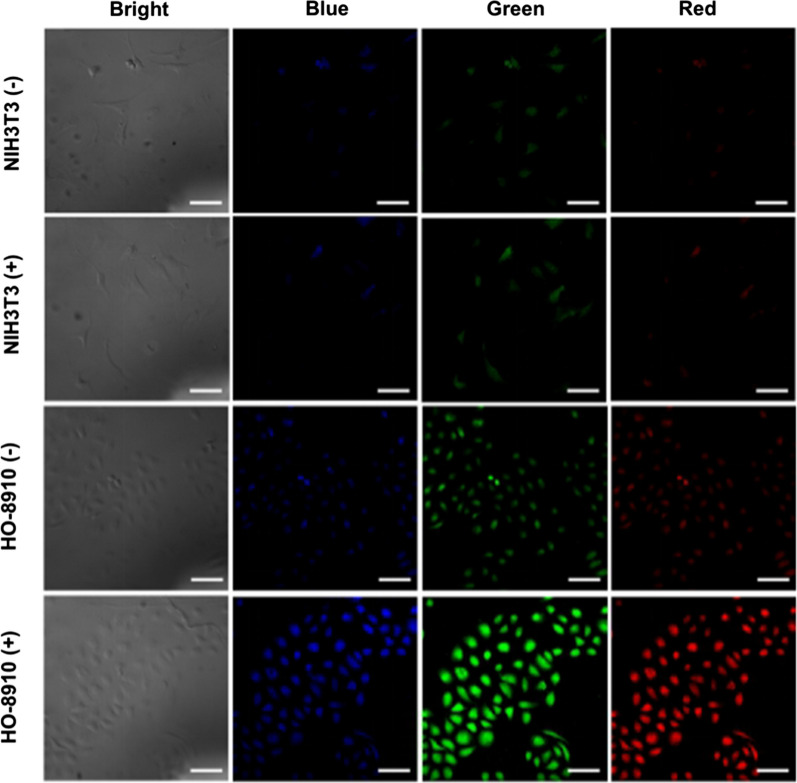
Multicolor (blue, green, red) fluorescence imaging of HO-8910 and NIH3T3 cells treated with Mn-N-CNSs@Anti-HE4 ( +) and Mn-N-CNSs (−), scale bar = 50 μm

To investigate the feasibility of Mn-N-CNSs@Anti-HE4 nanoprobe for targeted cellular imaging, HE4 positive HO-8910 cells and negative NIH3T3 cells were co-cultured with Mn-N-CNSs@Anti-HE4 and Mn-N-CNSs nanoprobes. As illustrated in Fig. 6, the NIH3T3 cells exhibited relatively weak multi-color fluorescence (blue, green, red) after the co-incubation with Mn-N-CNSs@Anti-HE4 and Mn-N-CNSs and there was no obvious difference between the two groups, indicating that the Anti-HE4 mAb didnot result in any facilitation in the cellular uptake of nanoprobe by NIH3T3 cells owing to the low HE4 expression. On the other hand, the HO-8910 cells also revealed slightly weak fluorescence signal under the incubation of Mn-N-CNSs nanoparticles. However, in the fluorescence image, the fluorescence signals were more intense and significantly enhanced in the HO-8910 cells treated by Mn-N-CNSs@Anti-HE4 compared to the Mn-N-CNSs treated group. It confirmed the targeting of Anti-HE4 mAb on the surface of Mn-N-CNSs@Anti-HE4 to the HO-8910 cells, therefore, facilitating the cellular fluorescence imaging to the ovarian cancer cells. Particularly, the cells remained their living morphology during the test period, indicating the low toxicity of Mn-N-CNSs@Anti-HE4 to the cells. Therefore, the Mn-N-CNSs@Anti-HE4 nanoprobe could effectively and selectively label the ovarian cancer cells with multi-color fluorescence.

**Fig. 7:**
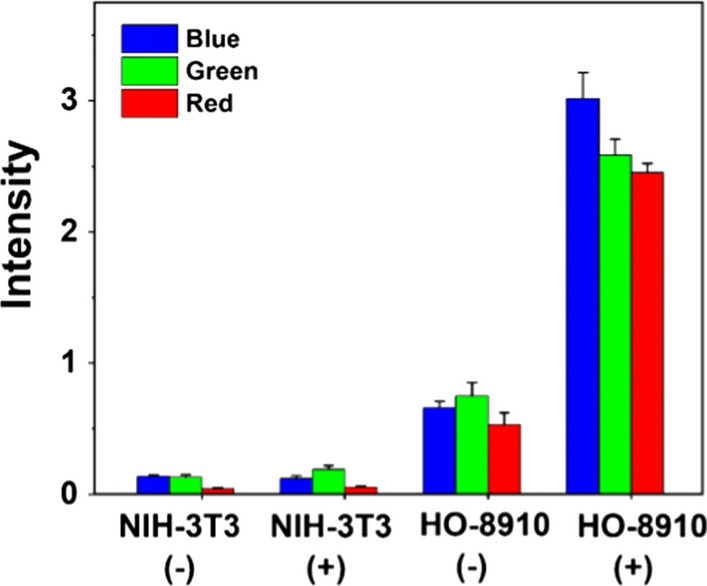
Multicolor (blue, green, red) fluorescent signal intensity of HO-8910 and NIH3T3 cells treated with Mn-N-CNSs@Anti-HE4 ( +) and Mn-N-CNSs (−)

The quantitative cellular uptake of the Mn-N-CNSs@Anti-HE4 by NIH3T3 and HO-8910 cells upon measuring the fluorescent intensity was shown in Fig. 7. It is obvious that the fluorescent intensity of the HO-8910 cells was tenfold higher than that of NIH3T3 cells in multi-colors. In addition, the Mn-N-CNSs@Anti-HE4 displayed considerably higher intensity than that of Mn-N-CNSs group. These findings confirmed the assistance of Anti-HE4 in the cellular uptake of Mn-N-CNSs by HO-8910 ovarian cancer cells, indicating the role of Anti-HE4 antibody in cellular uptake through active targeting via receptor-mediated endocytosis.

### In vitro enhanced MR imaging

**Fig. 8:**
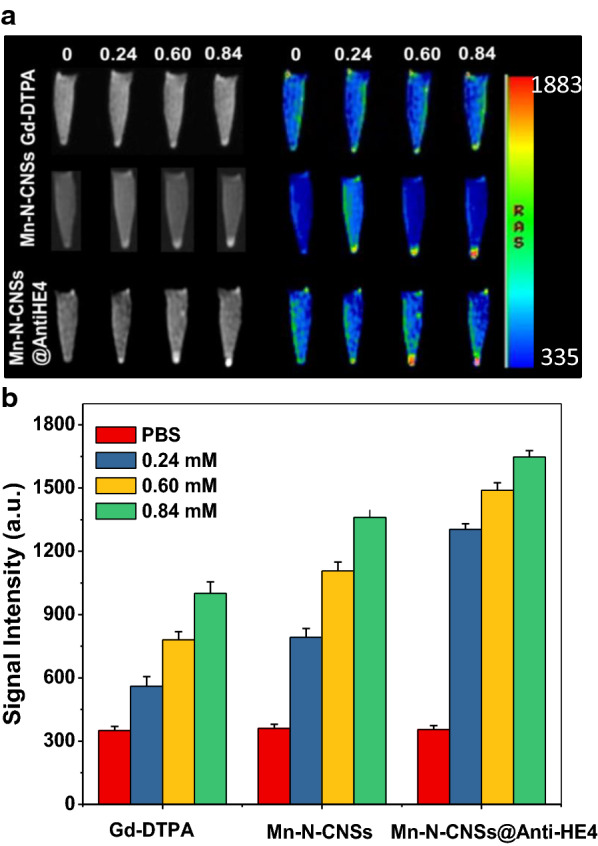
In vitro MR imaging of HO-8910 cells after treated with Gd-DTPA, Mn-N-CNSs and Mn-N-CNSss@Anti-HE4 at various concentrations: **a** T_1_-weighted and T_1_-map images, **b** MR signal intensities

Due to the superior MR contrast of the nanoprobes, an in vitro targeted MRI was performed. The HO-8910 and NIH3T3 cells were co-cultured with Mn-N-CNSs@Anti-HE4, Mn-N-CNSs, Gd-DTPA and PBS. As shown in Fig. 4C (a), the nanoprobe imaged cells could be identified on the basis of the bright signal at the tube bottom. Owing to the nonspecific absorption, the nanoprobes induced slight MR signal enhancement to the NIH3T3 cells compared with the control group. For NIH3T3 cells, the Anti-HE4 mAb didnot result in any enhancement in MR signal compared with Mn-N-CNSs attributed to the low affinity of Anti-HE4 mAb to NIH3T3 normal cells. On the other hand, the HO-8910 cells also revealed slightly weak MR signal under the incubation of Mn-N-CNSs. However, although the nonspecific absorption existed in the NIH3T3 cells and Mn-N-CNSs nanoprobe incubated HO-8910 cells, a notable brightening effect in the T_1_ MRI signal could still be distinguished for the Mn-N-CNSs@Anti-HE4 treated HO-8910 cells. The MR intensity values of different groups were presented in Fig. 4C (b). The MR signal intensity value of Mn-N-CNSs@Anti-HE4 in HO-8910 cells was higher than that of the NIH3T3 cells and Mn-N-CNSs in HO-8910 cells. It suggested that the Anti-HE4 mAb may favor the Mn-N-CNSs@Anti-HE4 to be internalized by the HE4 high expressed HO-8910 ovarian cancer cells, thus inducing a higher T_1_ signal. In addition, as shown in Fig. 8, the Mn-N-CNSs@Anti-HE4 exhibited overwhelming high MR signal in the HO-8910 cells compared with the commercial Gd-DTPA in the same Mn/Gd concentration, indicating the superiority of Mn-N-CNSs@Anti-HE4 as a MRI nanoprobe. These results demonstrated that the Mn-N-CNSs@Anti-HE4 could specifically and efficiently label the HO-8910 ovarian cancer cells both by fluorescence imaging and MR imaging. Therefore, the proposed Mn-N-CNSs@Anti-HE4 nanoprobe would favor targeted FL/MR dual-modal imaging for specific and accurate ovarian carcinoma diagnosis.

### In vivo targeted MR imaging

**Fig. 9:**
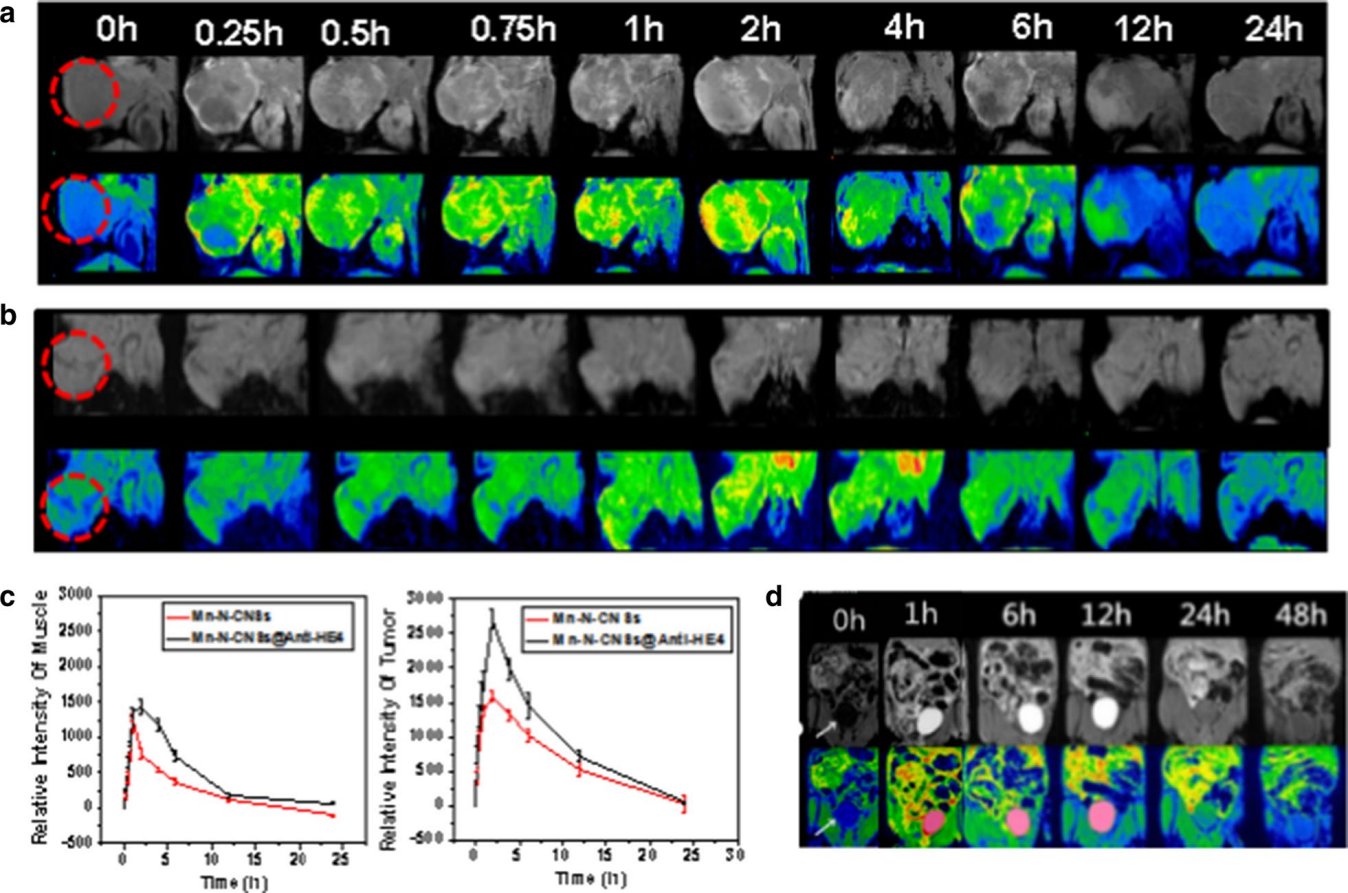
T1-weighted MRI (top) and corresponding pseudo-color images (bottom) of mice pre- and post-injection of (**a**) Mn-N-CNSs@Anti-HE4 nanoprobe and (**b**) Mn-N-CNSs at different time points. The inside of the red circles are tumors. **c** Signal intensity in the tumors and muscles. **d** T_1_-weighted and corresponding pseudo-color MR images of mice pre- and post-injection of Mn-N-CNSs@Anti-HE4. The white arrows indicate gall bladder

Encouraged by the excellent performance of in vitro MR imaging, we next investigated the ability of molecular MRI with the Mn-N-CNSs@Anti-HE4 nanoprobe in specifically imaging the ovarian carcinoma in HO-8910-tumor-bearing mice. The in vivo T_1_-weighted MR images of the mice were obtained pre- and post-injection of the Mn-N-CNSs@Anti-HE4 and Mn-N-CNSs at various time points. As shown in Fig. 9, it was clearly observed that the subcutaneous tumor area gradually brighter than the surrounding tissues after intravenous injection of Mn-N-CNSs@Anti-HE4 and Mn-N-CNSs nanoprobe. The T_1_ MR signal gradually increased within 2 h, then turned weaker over time. In comparison, the T_1_ signal in tumor region was significantly enhanced at the same time points after administration of Mn-N-CNSs@Anti-HE4. It indicated that the Anti-HE4 mAb could improve the targeting ability of the nanoprobe to the HO-8910 tumor to enhance the MR contrast effect in vivo. Besides, as shown in Fig. 9d, bright T1 MR signal was found in the gall bladder within 1 h administration of Mn-N-CNSs@Anti-HE4 nanoprobe and the signal then disappeared after 24 h, indicating the prepared nanoprobe was mainly cleared from body by the urinary system. The urine samples from the mice after the injection were collected for further investigation. The nanoparticles were harvested by centrifugation, and analysis on the fragment found strong T1-weighted signals, along with strong FL that is characteristic of Mn-N-CNSs (Additional file 1: Fig. S6). These results suggest that the Mn was still well incorporated into nanoparticles during excretion, thus ensuring the stability of the MR signal and low toxicity.

### In vivo biocompatibility analysis

**Fig. 10:**
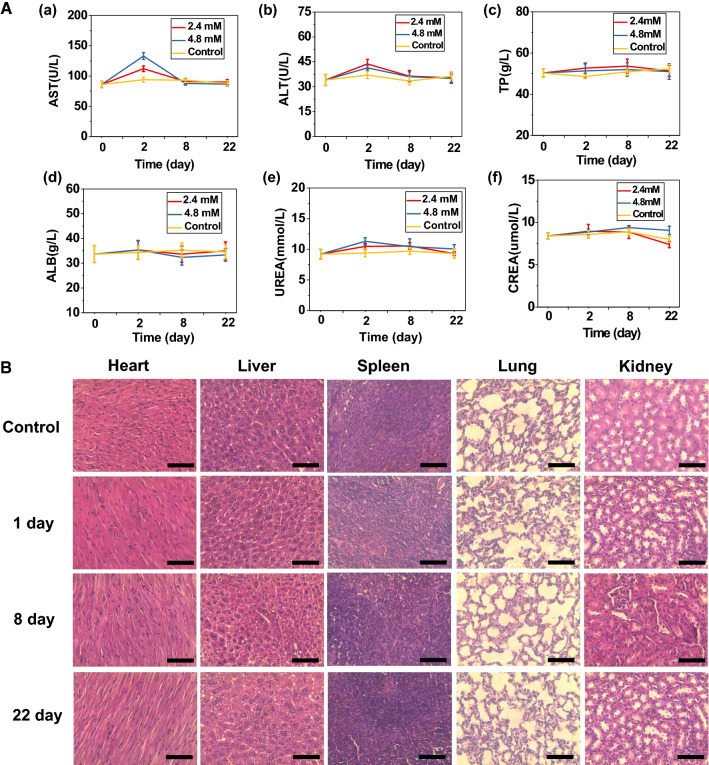
In vivo toxicity test of Mn-N-CNSs@Anti-HE4: (**A**, **a**–**f**) Mouse serum biochemistry analysis before (0 day, control) and after injection of Mn-N-CNSs@Anti-HE4 (2.4 mM and 4.8 mM) for 1, 8, 22 days. **B** Histological images of the heart, liver, spleen lung, and kidneys of mice 1, 8 and 22 days post-intravenous injection of Mn-N-CNSs@Anti-HE4 (4.8 mM) and in control mice. The organs were sectioned and stained with hematoxylin and eosin (H&E) and observed under a light microscope, scale bar = 100 μm

In vivo biocompatibility assays of Mn-N-CNSs@Anti-HE4 were conducted on healthy Kunming mice model for 22 days. Mn-N-CNSs@Anti-HE4 were intravenously injected into the mice and the saline injected mice were refered as control group. At determined time points, blood was collected for the complete blood count and serum biochemistry tests. For the complete blood count assay, several important standard markers including red blood cells (RBC), white blood cells (WBC), hematocrit (HCT), hemoglobin (HGB) platelet (PTL) and mean corpuscular volume (MCV) were selected to investigate the effect to immune system. As indicated in Additional file 1: Fig. S7, slight variations could be observed in the values of various hematological markers after 1d administrations. However, the values returned back to the normal level of control animals after 8 days. Though the values of hematological markers fluctuated in a short period after administration of Mn-N-CNSs@Anti-HE4 nanoprobe, the values were still within the normal ranges for each markers. The serum biochemistry study was also carried out to monitor the potential toxic effect of Mn-N-CNSs@Anti-HE4 nanoprobe. Indicators of heart, kidney and liver functions were evaluated including aspartate aminotransferase (AST), alanine aminotransferase (ALT), total protein (TP), indicators-albumin (ALB), total protein (TP) and creatinine. As shown in Fig. 10a, the values of various biochemical markers revealed a higher level for the animals treated by Mn-N-CNSs@Anti-HE4 nanoprobe compared with the saline treated group at 1 day post-injection. Then, the values lowered back to the normal level after 8 day post-injection, which was consistent with the fluctuation behavior of hematological markers. These results demonstrated that the Mn-N-CNSs@Anti-HE4 nanoprobe injection may slightly affect the biological conditions of the mice in a short period, but without acute damage in a long term.

Besides, the biosafety of the nanoprobe was further investigated by the histological analysis. Major organs (heart, kidney, liver, spleen and lung) were sliced and stained for the detailed microscopic evaluation of the interaction of tissues and Mn-N-CNSs@Anti-HE4 nanoprobe. H&E-stained images of organ sections displayed no apparent inflammation, histopathological abnormalities or lesions after treatment with the probe. The body weights of the mice were also monitored during the whole test period. The mice upon Mn-N-CNSs@Anti-HE4 administration exhibited no notable variation compared to those of the control group in a long term (Additional file 1: Fig. S8). These results demonstrated the low toxicity and good biocompatability of the Mn-N-CNSs@Anti-HE4 nanoprobe, unveiling its further applications in biomedical fields.

## Conclusions

In summary, we successfully synthesized a precise diagnostic nanoprobe based on Mn-N-CNSs@Anti-HE4, affording MR imaging modality, in addition to inherent fluorescence imaging. The prepared Mn-N-CNSs@Anti-HE4 nanoprobe revealed excellent aqueous dispersity, good colloidal stability. The Mn-N-CNSs@Anti-HE4 nanoprobe holds a tunable photoluminiscnce property, which favors the highly specific and sensitive multi-color fluorescence imaging of ovarian carcinoma cells. More importantly, the Mn-N-CNSs@Anti-HE4 nanoprobe afforded high longtudinal r_1_ relaxivity, providing good contrast effect for the HO-8910 ovarian tumor in vitro and in vivo. In vitro FL/MR imaging and in vivo MR imaging results indicated Mn-N-CNSs@Anti-HE4 nanoprobe were promising candidate for ovarian cancer cells targeted labeling and imaging. No apparent toxicity of Mn-N-CNSs@Anti-HE4 nanoprobe was observed for the in vitro cytotoxicity and in vivo serum biochemistry and histological analysis study. On the basis of the results demonstrated in this work, the biocompatible nanoprobe with FL/MR imaging capacity hold great promise in bioimaging applications. However, work on the development of Mn-N-CNSs-based nanoprobes for in vivo FL/MR dual-modal imaging is still in its preliminary stages due to the maximum blue light emission of Mn-N-CNSs. Tuning the photoluminescence of Mn-N-CNSs into the near-infrared region and simultaneously achieving a high fluorescence quantum yield will be necessary to accelerate the application of Mn-N-CNSs@Anti-HE4 in targeted FL/MR dual-modal imaging of ovarian carcinoma.

## Supplementary information

**Additional file 1: Figure S1.** Optimization of reaction conditions. **Figure S2.** Typical EDS pattern of Mn-N-CNSs. **Figure S3.** Stability of the Mn-N-CNSs in different biological fluids. **Figure S4.** The fluorescence stability of Mn-N-CNSs. **Figure S5.** Effect of pH (A) and ionic strengths (B) on the fluorescence intensity of Mn-N-CNSs. **Figure S6.** Fluorescence (A) and T1-weighted images as well as T1 relaxation time (B) of urine samples, taken ~ 12 h after the injection of Mn-N-CNSs@Anti-HE4. **Figure S7.** Complete blood count study of the mice treated by Mn-N-CNSs@Anti-HE4 at various concentraions for 1, 8, 22 days. **Figure S8.** Body weight changes of the mice after the administration of Mn-N-CNSs and saline for different days.

## Data Availability

All data generated or analysed during this study are included in this published article and its Additional file 1.
