# Supplementary material for: Development of fluorescence/MR dual-modal manganese-nitrogen-doped carbon nanosheets as an efficient contrast agent for targeted ovarian carcinoma imaging
Source: J Nanobiotechnology. 2020 Nov 30;18:175. doi: 10.1186/s12951-020-00736-w (PMC7708123; doi:10.1186/s12951-020-00736-w)
Supplement: Supplementary file 1 — Additional file 1: Figure S1. Optimization of reaction conditions. Figure S2. Typical EDS pattern of Mn-N-CNSs. Figure S3. Stability of the Mn-N-CNSs in different biological fluids. Figure S4. The fluorescence stability of Mn-N-CNSs. Figure S5. Effect of pH (A) and ionic strengths (B) on the fluorescence intensity of Mn-N-CNSs. Figure S6. Fluorescence (A) and T1-weighted images as well as T1 relaxation time (B) of urine samples, taken ~ 12 h after the injection of Mn-N-CNSs@Anti-HE4. Figure S7. Complete blood count study of the mice treated by Mn-N-CNSs@Anti-HE4 at various concentraions for 1, 8, 22 days. Figure S8. Body weight changes of the mice after the administration of Mn-N-CNSs and saline for different days. [file 12951_2020_736_MOESM1_ESM.docx]

**Additional file 1**

**Development of fluorescence/MR dual-modal manganese-nitrogen-doped carbon nanosheets as an efficient contrast agent for targeted ovarian carcinoma imaging**

Cuiping Han^1,2^ ^†*^, Ting Xie ^1†^, Keying Wang^3†^, Shang Jin^1^, Ke Li^1^, Peipei Dou^1^, Nana Yu^1^, Kai Xu^2*^

^1^ School of Medical Imaging, Xuzhou Medical University, Xuzhou, 221006, China

^2^ Department of Radiology, Affiliated Hospital of Xuzhou Medical University, Xuzhou, 221004, China

^3^ Department of Medical imaging, Jinshan Hospital Affiliated to Fudan University, Shnghai, 200540, China

^†^Cuiping Han, Ting Xie and Keying Wang contributed equally to this work.

^*^Corresponding authors: [hancp@xzhmu.edu.cn](mailto:hancp@xzhmu.edu.cn) and [xkpaper@163.com](mailto:xkpaper@163.com)

**Fig. S1** Optimization of reaction conditions.The reaction time (A) and temperature (B) on the fluorescence spectra of Mn-N-CNSs. The effect of different molar ratios of Mn:DTPA on the fluorescence intensity (C) and MRI relaxation properties of Mn-N-CNSs (D).

**Fig. S****2** Typical EDS pattern of Mn-N-CNSs.

**Fig. S3** (A) Stability of the Mn-N-CNSs in ID water, PBS, FBS, and RPMI-1640. (B) DLS analysis of the Mn-N-CNSs in ID water, PBS, FBS, and RPMI-1640, respectively. (C) T1-weighted MR images of Mn-N-CNSs in ID water, PBS, FBS, and RPMI-1640, respectively.

**Fig. S4** The fluorescence stability of Mn-N-CNSs. (A) Effect of storage time on fluorescence spectra (a) and fluorescence intensity (b) of Mn-N-CNSs solution. (B) Fluorescence spectra (a) and fluorescence intensity (b) intensity change of Mn-N-CNSs under exposure to the UV light for various time.

**Fig. S5** Effect of pH (A) and ionic strengths (B) on the fluorescence intensity of Mn-N-CNSs. (Ionic strengths were controlled by various concentrations of NaCl).

**Fig. S6** Fluorescence (A) and T1-weighted images as well as T1 relaxation time (B) of urine samples, taken ~12 h after the injection of Mn-N-CNSs@Anti-HE4. PBS solution used as a control in MR imaging experiments.

**Fig. S7** Complete blood count study of the mice treated by Mn-N-CNSs@Anti-HE4 at various concentraions for 1, 8, 22 days.

**Fig. S8** Body weight changes of the mice after the administration of Mn-N-CNSs@Anti-HE4 and saline for different days.
